# Hyperinfectivity in Cholera: A New Mechanism for an Old Epidemiological Model?

**DOI:** 10.1371/journal.pmed.0030280

**Published:** 2006-06-27

**Authors:** Mercedes Pascual, Katia Koelle, Andrew P Dobson

**Affiliations:** **1**Department of Ecology and Evolutionary BiologyUniversity of MichiganAnn Arbor, MichiganUnited States of America; **2**Center for Infectious Disease DynamicsPennsylvania State UniversityUniversity Park, PennsylvaniaUnited States of America; **3**Department of Ecology and Evolutionary BiologyPrinceton UniversityPrinceton, New JerseyUnited States of America

Hartley et al. [ 1] have recently proposed an epidemiological model for the dynamics of cholera that explicitly incorporates a hyperinfectious stage of the pathogen Vibrio cholerae, following laboratory findings that passage of the bacterium through the gastrointestinal tract results in a short-lived more highly infectious state. The paper and its commentary [ 2] emphasize that this model provides a basis for the transmission pathway known as “human-to-human” and demonstrates its importance, relative to the “environment-to-human” pathway, in the “explosive” character of cholera epidemics. Nevertheless, several important points seem to be missing from the discussion.

First, epidemiological models that treat transmission as “human-to-human” do exist in both the older literature [ 3] and in more recent cholera studies [ 4, 5]. The latter use time series models to explain the interannual dynamics of cholera outbreaks in endemic areas. Their application to the temporal patterns of cholera in both recent and historical records from Bangladesh show that the force of infection is clearly related to previous incidence levels, as expected for “human-to-human” transmission. This work has also shown that environmental variables (El Niño, rainfall) modulate this type of transmission [ 5], so that the focus on the environment is relevant even for the “human-to-human” pathway.

Second and more importantly, we can ask whether the model of Hartley et al. differs from the standard treatment of “human-to-human” transmission in epidemiological models. In particular, does the so-called “explosive” behavior differ from the well-known exponential growth of cases at the beginning of an epidemic, when there is little or no immunity built into the population? Inspection of the temporal scales involved in the dynamics tells us that this is not the case: we can collapse their treatment of transmission via a hyperinfectious stage into a more standard direct-transmission formulation. This is because the dynamics of the hyperinfectious stage in the environment are much faster than that of the number of cases, with the average lifespan of a hyperinfectious bacterium (their variable BHI) being on the order of 5 h, whereas an infected individual (their variable I) continues to shed for approximately 5 d. Therefore, to a first approximation, BHI sees I as “constant” for a sufficient length of time to reach “equilibrium” for any given value of I. It follows that this “equilibrium” concentration of the hyperinfectious stage in the environment effectively tracks the size of the infected population; in other words, BHI is simply proportional to I. Simulations of the model with the parameter values of the Hartley et al. paper confirm this expectation for the whole course of an epidemic. We can then get rid of this variable and write the transmission rate as a function of the number of susceptibles and the number of infected individuals, as is traditionally done in epidemiological models. Thus, for purposes of modeling cholera epidemics, we do not need to explicitly represent the hyperinfectious stage, unless the questions and mechanisms we are examining are specifically about this stage (as was the case in Hartley et al.), and “explosive” behavior does not refer to a different type of dynamics than that of standard models for human-to-human transmission.

There is another way, however, in which the epidemics may have been called “explosive” by Hartley et al.: the growth rate of the epidemics in their model is much higher when “human-to-human” transmission becomes dominant relative to “environment-to-human” transmission. This brings us to the important epidemiological quantity known as R0, which measures the number of secondary cases produced by an infected individual in a pool of susceptibles, that is, at the beginning of an outbreak. Hartley et al. report a new formula for cholera's R0 (Equation 4 in their paper). There is an interesting discrepancy between Hartley et al.'s R0 estimate when “human-to-human” is dominant (R0, ∼18) and the value we obtain for cholera data for Matlab, Bangladesh (R0, ∼3) (unpublished result). Our estimate is close to the values Hartley et al. propose when “environment-to-human” transmission is dominant, even though our estimate is obtained from a model of “human-to-human” transmission. As far as we can tell from the information provided, the derived expression for R0 in Hartley et al. is an approximation. It appears to hold exactly when the dynamics of both the hyperinfectious and the environmental stage occur on fast temporal scales, quickly “equilibrating” and tracking the number of cases. While this assumption, as we have argued, applies to the hyperinfectious stage, it does not to the environmental one, as demonstrated by similar model simulations. Hartley et al.'s expression for R0 would then overestimate the reproductive number of the disease, making it more explosive than it is (see Figure 4 in the paper).

The discrepancy in our estimates has an important consequence: while an epidemic declines from a depletion of susceptibles in the Hartley et al. model, the seasonal outbreaks we observe in Bangladesh are curtailed prior to a significant depletion of susceptibles [ 5]. This implies that the transmission rate must effectively be decreasing as the epidemic peaks. Indeed, recent observations of vibriophage dynamics in Bangladesh have given rise to the hypothesis that seasonal outbreaks may be self-limiting due to amplification of *Vibrio*-specific phage [ 6, 7]. The dynamics of phage predation are a likely mechanism for the observed reduction in cholera transmission rate at the end of seasonal outbreak.

Despite these differences, both our analyses and Hartley et al.'s model accentuate the need to consider some variant of “human-to-human” transmission to explain cholera dynamics. An important issue is therefore what we should call “human-to-human” transmission. Clearly, the categorization of the two routes of transmission (“human-to-human” and “environment-to-human”) is a simplification, albeit useful for the purpose of modeling the disease, that considers only the two extremes of a continuous axis defined by the strength of the feedback between (previous) cases and transmission rate and by the different temporal scales of transmission. For the “environment-to-human” type, this feedback is weak (in the extreme, nonexistent) as the bacterium concentration in the environment becomes dominated by its survival, population growth, environmental drivers, and the stochastic nature of these processes. At the other extreme, the feedback is strong and the transmission rate is a function of cases. This definition is more general and more practical than the one that restricts “human-to-human” transmission to that mediated by the hyperinfectious state. For issues of control, the more general definition appears more relevant, unless we are considering control measures that would specifically target the concentration of the hyperinfectious stage.

Many open questions remain on the modeling of cholera in connection to transmission pathways. For example, early-warning systems and associated predictive models for endemic and epidemic regions remain to be developed and tested. In contrast, the importance of sanitary conditions, sewage treatment, and clean water for cholera prevention and eradication has been known for a very long time. Nevertheless, cholera is today in its seventh pandemic and, as a “disease of poverty” [ 8], continues to represent a significant public health burden around the world. Neither an exclusive focus on the environment nor an emphasis on socio-economic factors alone is sufficient to address the cholera problem today. Developing a better mechanistic understanding of the factors that initiate, amplify, and defuse regular seasonal outbreaks in endemic areas and irregular “epidemic” outbreaks in others should prove valuable to develop viable control strategies for this and other enteric diseases.

## References

[pmed-0030280-b1] Hartley DM, Morris JG, Smith DL (2006). Hyperinfectivity: A critical element in the ability of
V. cholerae to cause epidemics?. PLoS Med.

[pmed-0030280-b2] Codeco CT, Coelho FC (2006). Trends in cholera epidemiology. PLoS Med.

[pmed-0030280-b3] Cvjetanovic B (1982). The dynamics of bacterial infections. Population dynamics of infectious diseases, theory, and applications.

[pmed-0030280-b4] Koelle K, Pascual M (2004). Disentangling extrinsic from intrinsic factors in disease dynamics: A nonlinear time series approach with an application to cholera. Am Naturalist.

[pmed-0030280-b5] Koelle K, Rodo X, Pascual M, Yunus M, Mostafa G (2005). Refractory periods to climate forcing in cholera dynamics. Nature.

[pmed-0030280-b6] Faruque SM, Naser IB, Islam MJ, Faruque AS, Ghosh AN (2005). Seasonal epidemics of cholera inversely correlate with the prevalence of environmental cholera phages. Proc Natl Acad Sci U S A.

[pmed-0030280-b7] Faruque SM, Islam MJ, Ahmad QS, Faruque AS, Sack DA (2005). Self-limiting nature of seasonal cholera epidemics: Role of host-mediated amplification of phage. Proc Natl Acad Sci U S A.

[pmed-0030280-b8] Bonilla-Castro E, Rodriguez P, Carrasquilla G (2000). La enfermedad de la pobreza: El colera en los tiempos modernos.

